# Epidemiological Survey-Based Formulae to Approximate Incidence and Prevalence of Neurological Disorders in the United States: a Meta-Analysis

**DOI:** 10.1371/journal.pone.0078490

**Published:** 2013-10-24

**Authors:** Cesar V. Borlongan, Jack Burns, Naoki Tajiri, Christine E. Stahl, Nathan L. Weinbren, Hideki Shojo, Paul R. Sanberg, Dwaine F. Emerich, Yuji Kaneko, Harry R. van Loveren

**Affiliations:** Department of Neurosurgery and Brain Repair, University of South Florida, Tampa, Florida, United States of America; INRCA, Italy

## Abstract

**Background:**

This study aims to create a convenient reference for both clinicians and researchers so that vis-à-vis comparisons between brain disorders can be made quickly and accurately. We report here the incidence and prevalence of the major adult-onset brain disorders in the United States using a meta-analysis approach.

**Material and Methods:**

Epidemiological figures were collected from the most recent, reliable data available in the research literature. Population statistics were based on the most recent census from the US Census Bureau. Extrapolations were made only when necessary. The most current epidemiological studies for each disorder were chosen. All effort was made to use studies based on national cohorts. Studies reviewed were conducted between 1950 and 2009. The data of the leading studies for several neurological studies was compiled in order to obtain the most accurate extrapolations. Results were compared to commonly accepted values in order to evaluate validity.

**Results:**

It was found that 6.75% of the American adult population is afflicted with brain disorders. This number was eclipsed by the 8.02% of Floridians with brain disorders, which is due to the large aged population residing in the state.

**Conclusions:**

There was a noticeable lack of epidemiological data concerning adult-onset brain disorders. Since approximately 1 out of every 7 households is affected by brain disorders, increased research into this arena is warranted.

## Introduction

Aging, especially within the brain, has been a topic of major importance to the field of public health. Age-related brain disorders will assuredly increase dramatically in the United States over the coming decades due to the aging baby boomer generation (individuals born 1946-1964). The most common age-related brain disorders include amyotrophic lateral sclerosis (ALS), Alzheimer’s disease, brain tumor, epilepsy, HIV dementia, Huntington’s disease, multiple sclerosis, Parkinson’s disease, stroke, and traumatic brain injury (TBI). Among the more common epidemiological statistics often quoted are incidence and prevalence. Incidence is defined as the annual number of individuals that have been diagnosed with a particular disorder, while prevalence describes the number of individuals that currently have a particular disorder. A review of the scientific literature over the past ten years indicates that the incidence and prevalence of adult-onset brain disorders have not been explicitly stated together as a group. Rather, the incidence and prevalence are often mentioned in the introduction section of many research articles dedicated for each specific brain disorder, thereby making vis-à-vis comparisons between diseases overly cumbersome. Thus, a reference guide summarizing the most current incidence and prevalence of the most common aging-related brain disorders is lacking. This study provides the most current, well-supported data regarding the incidence and prevalence of the major adult-onset brain disorders in the United States using a meta-analysis approach [1]. Our review of prevalence and incidence data for brain disorders in the United States [2-27] revealed that epidemiological data in the United States are extremely scarce for a number of adult-onset brain disorders. This is surprising because up-to-date national studies exist in Australia and many European countries [28,29]. This study provides an in-depth analysis of the state of Florida. Simple calculations have been provided so that the data can be adjusted for any given American state. 

## Material and Methods

An analysis of epidemiological studies was performed to obtain the most recent and reliable incidence and prevalence rates for the most common adult-onset brain disorders. This study adhered to the meta-analysis of observational studies in epidemiology (MOOSE) guidelines [1] (See also Checklist S1). The search terms included each specific adult brain disorder, incidence, prevalence, and United States. When possible, incidence and prevalence figures were extracted directly from the literature [2-27]. Data were derived from PUBMED and Google in generating the incidence and prevalence rates provided in number of diseased individuals per 100,000 individuals. Only the most current data from epidemiology studies were used for each disorder. While PubMed was used to search scholarly records, Google was used to search a wider variety of more publicly-available sources that better reflect the population. For example, an incidence rate of 2.2 indicates that 2.2 individuals out of every 100,000 persons are diagnosed with a condition annually. In order to find the incidence and prevalence nationwide, the incidence and prevalence rates were extrapolated using the US Census 2009 population estimate. The following calculation demonstrates this extrapolation:

(Incidence Rate / 100,000 individuals) * Population Estimate = Incidence

(Prevalence Rate / 100,000 individuals) * Population Estimate = Prevalence

As previously mentioned, incidence is the annual number of individuals that have been diagnosed with a disorder and prevalence is the number of individuals that currently have a disorder. Therefore, an interesting relationship exists between the two epidemiological statistics. This relationship states that the prevalence of a disorder is equal to the incidence of that disorder multiplied by the mean duration of the disease. The following calculation more explicitly states this relationship:

Incidence * Mean Duration of Disorder = Prevalence

The incidences of Alzheimer’s disease, epilepsy, multiple sclerosis, Parkinson’s disease, and TBI were directly cited in literature. The ALS incidence was obtained by multiplying the incidence rate by the 2009 population estimate, as stated in the calculation method described above. The same procedure was used to obtain the incidences of brain tumor and stroke. The incidence rate for Huntington's disease was obtained by taking the arithmetic average of the two most commonly cited incidence rates in scientific literature. This calculated incidence rate was multiplied by the 2009 population estimate to obtain the actual incidence. Finally, the incidence of HIV dementia was obtained by first finding the incidence of HIV, which was found by multiplying the incidence rate and the 2009 population estimate. Since HIV dementia only affects a certain percentage of those with HIV, the HIV incidence was multiplied by the percentage of those with HIV and dementia in order to find the incidence of HIV dementia.

Similarly, the prevalence of Alzheimer’s disease, epilepsy, multiple sclerosis, stroke, and TBI was directly cited in literature. The prevalence of ALS was obtained using a multi-step process. First, the incidence of ALS was calculated by multiplying the incidence per 100,000 individuals by the 2009 population estimate. Next, the calculated incidence was then multiplied by the mean duration of the disease in order to find the prevalence of ALS. The brain tumor prevalence was obtained by multiplying the prevalence rate by the 2009 population estimate. The prevalence rate for Huntington's disease was obtained by taking the arithmetic average of the six most commonly cited prevalence rates. This calculated prevalence rate was multiplied by the 2009 population estimate to obtain the prevalence of Huntington’s disease. The incidence of HIV dementia was also obtained in a multi-step process. First, the prevalence of HIV was found by multiplying the prevalence rate by the 2009 population estimate. Again, HIV dementia only affects a certain percentage of those with HIV. As such, the HIV prevalence was multiplied by the ratio of HIV patients with dementia, which is cited in the literature, in order to find the prevalence of HIV dementia. The figure for Parkinson’s disease was calculated by multiplying the percentage of Americans with the disease by the 2009 population estimate.

In order to estimate the percentage of adults and households affected with brain disorders, statistical data on the United States’ population were analyzed. The totals for population and households in the United States were obtained using US Census 2009 estimates. The adult population (individuals 18 years of age and older) was also obtained using the 2005 US Census estimates The estimated number of adults with brain disorders was calculated by summing the prevalence of each of the major adult-onset brain disorders. The percentage of the United States adult population afflicted with brain disorders was obtained by dividing the estimated number of adults with brain disorders by the 2009 US population estimate. The following equation clearly demonstrates this calculation:

Adults in US with Brain Disorders / Adult Population in US = Percentage of Adults in US with Brain Disorders

Similarly, the percentage of households affected by brain disorders was obtained by dividing the number of adults with brain disorders by the estimated number of US households, or more explicitly:

Adults in US with Brain Disorders / US Households = Percentage of US Households Affected by Brain Disorders

In order to obtain state-by-state data, the national data figures were simply scaled down to state levels based on population ratios. It should be noted, however, that state data do exist for the prevalence of Alzheimer’s disease. For the sake of accuracy, these prevalence figures for Alzheimer’s disease were used directly rather than extrapolated.

The incidences of disorders in the state of Florida were obtained by multiplying the United States’ incidence data by Florida’s percentage of the entire United States population. The following equation accurately demonstrates this relationship:

(Adult Population in Florida / Adult Population in US) * US Incidence = Incidence of Disorder in Florida

As previously mentioned, the prevalence for Alzheimer’s disease is directly cited in the literature and is based on a 2005 estimate. The prevalence of the remaining brain disorders was obtained by multiplying the United States prevalence data by Florida’s percentage of the entire United States population. The prevalence was obtained by using the following equation:

(Adult Population in Florida / Adult Population in US) * US Prevalence = Prevalence of Disorder in Florida

Data from the US Census were used in order to estimate the number of Floridians affected by brain disorders. The total population of Florida was obtained by using the 2009 US Census estimates. The adult population was also obtained using US Census estimates, but the estimate was based off of 2008 data. The total number of Florida households was found in the 2000 US Census. The estimated number of adults with brain disorders in Florida was calculated by summing the prevalence of each of the major adult-onset brain disorders in the state of Florida. These prevalence statistics included both the cited Alzheimer’s disease data and the prevalence data that were extrapolated using the methods described above. The percentage of the Florida adult population with brain disorders was obtained by dividing the estimated number of adults with brain disorders by the 2009 Florida population estimate, or:

Adults in Florida with Brain Disorders / Adult Population in Florida = Percentage of Adults in Florida with Brain Disorders

The percentage of households in Florida affected with brain disorders was obtained by dividing the number of adults with brain disorders by the estimated number of Florida households. The following equation describes this derivation:

Adults in Florida with Brain Disorders / Florida Households = Percentage of Florida Households Affected by Brain Disorders

While this study focused on the state of Florida, the same procedures can be used to estimate the prevalence, incidence, and percentage of those affected by brain disorders for any state in America. The following equations describe how to obtain figures of interest:

(Adult Population in State / Adult Population in US) * US Incidence = Incidence of Disorder in State

(Adult Population in State / Adult Population in US) * US Prevalence = Prevalence of Disorder in State

Adults in State with Brain Disorders / Adult Population in State = Percentage of Adults in State with Brain Disorders

Adults in State with Brain Disorders / State Households = Percentage of State Households Affected by Brain Disorders

## Results

**Figure 1:**
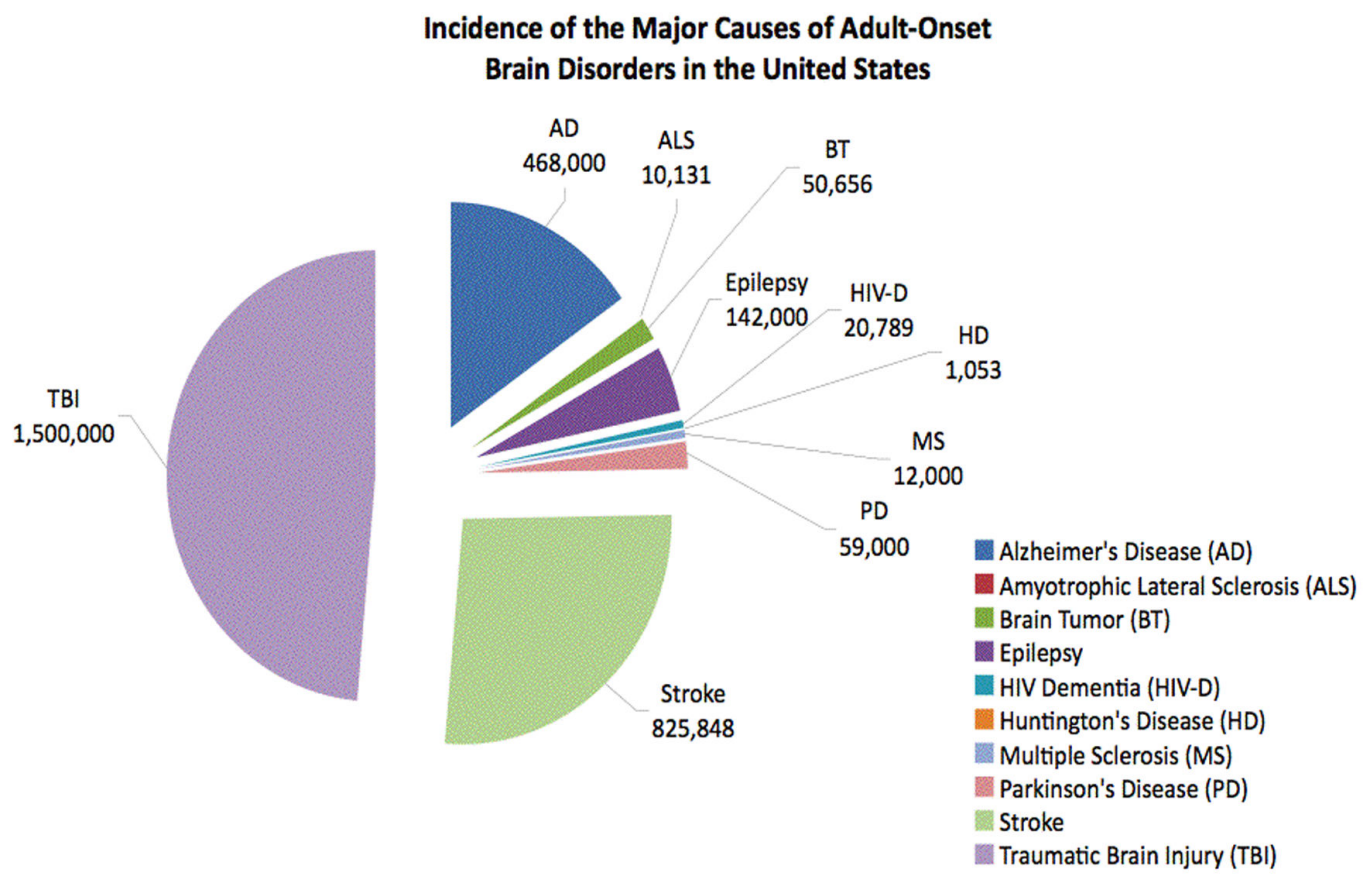
Most current incidence data of adult-onset brain disorders for the United States.

The incidences of the most common adult-onset brain disorders in the United States were obtained from sources that have been published during the past decade with the exception of ALS, Huntington’s disease, and TBI (Table 1). All effort was made to locate the most accurate and recent data. Table 2 and Figure 1 summarize the incidence data for brain disorders in the United States. 

**Table 1:** Explanation of Sources.

**Author/Organization**	**Year**	**Topic**
Annegers et al.	1991	ALS
Centers for Disease Control (CDC)	2008	HIV Prevalence in the US
Centers for Disease Control (CDC)	2008	Stroke Prevalence in the US
Central Brain Tumor Registry of the United States	2006	Brain Tumor
Cronin et al.	2007	ALS
Day	1996	Population Household Estimates
de Lau and Breteler	2006	Parkinson’s disease
Eisen et al.	2004	ALS
Folstein et al.	1987	Huntington’s disease
Hall et al.	2008	HIV in the US
Herbert et al.	2004	Alzheimer’s disease
Hirtz et al.	2003	Neurologic Disorders
Kokman et al.	1994	Huntington’s disease
Kurland et al.	1958	Neurologic Disorders
Kurtzke and Kurland	1983	Neurologic Disorders
Myrianthopoulos	1973	Huntington’s disease
Pearson et al.	1955	Huntington’s disease
Population Estimates Program	2009	Population Estimates by Residence
Population Estimates Program	2009	Population Estimates by Sex and Age for 2000-2008
Population Estimates Program	2009	Population Estimates by Sex and Age for 2008
Roos et al.	1993	Huntington’s disease
Sacktor et al.	2002	HIV
Thurman et al.	1999	TBI in the US
U.S. Census Bureau Population Division	2009	Florida Population Estimates
Williams et al.	2001	Stroke in the US
Zaloshnja et al.	2005	TBI Prevalence US

**Table 2:** Incidence of the major causes of adult-onset brain disorders in the United States.

Diagnosis/Cause	People Diagnosed Annually
Alzheimer's Disease	468,000 [2]
Amyotrophic Lateral Sclerosis	10,131 [3]
Brain Tumor	50,656 [4]
Epilepsy	142,000 [2]
HIV Dementia	20,789 [5,6]
Huntington's Disease	1,053 [7,8]
Multiple Sclerosis	12,000 [2]
Parkinson's Disease	59,000 [2]
Stroke	825,848 [9]
Traumatic Brain Injury	1,500,000 [10]
**Total Estimated Incidence**	**3,089,477**

**Figure 2:**
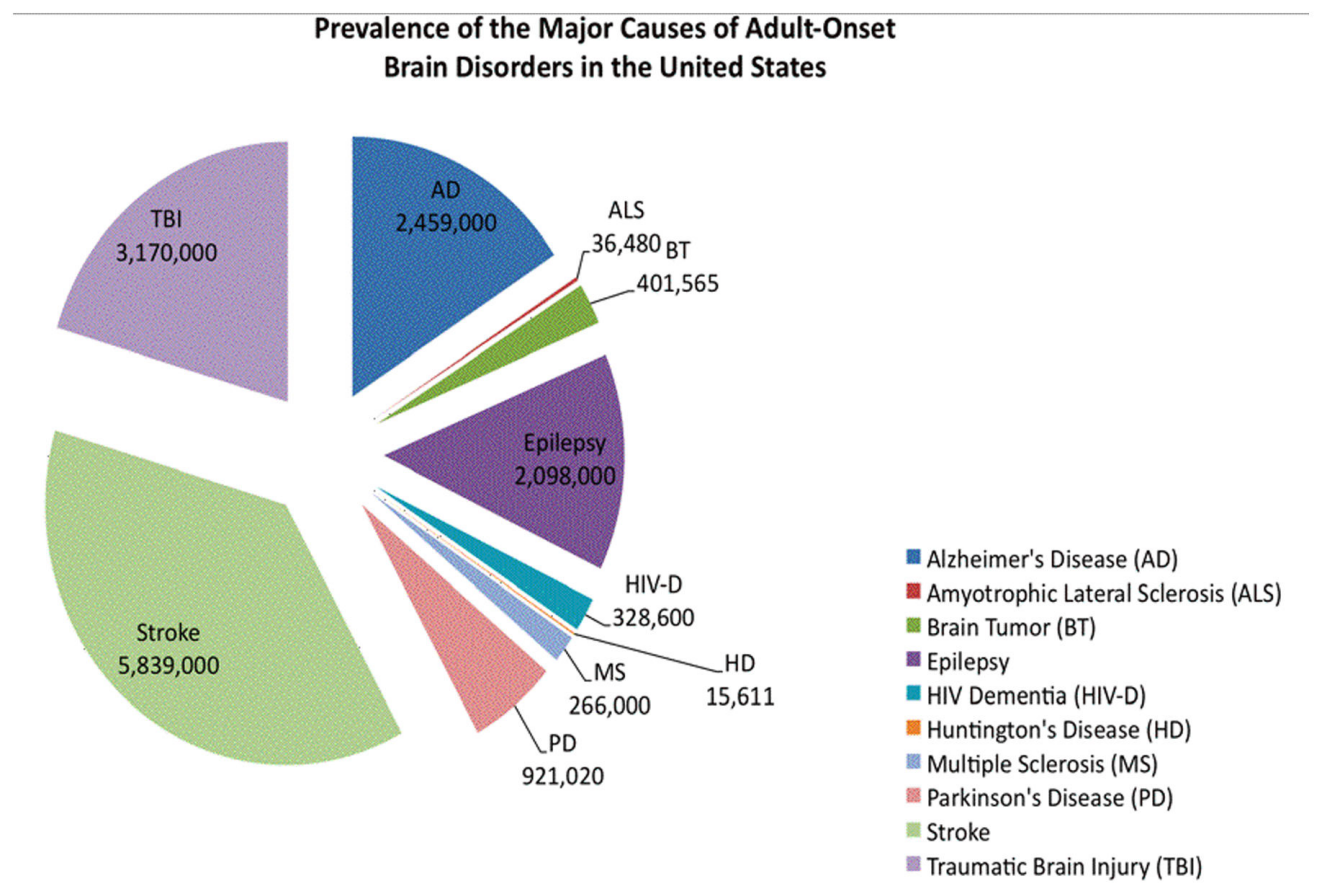
Most current prevalence data of adult-onset brain disorders for the United States.

The prevalence figures were obtained from studies conducted within the past ten years with the exception of Huntington’s disease. The prevalence of Huntington’s disease was obtained from sources published between 1955 and 1994. The prevalence of the most common brain disorders is given in Table 3 and Figure 2.

**Table 3:** Prevalence of the major causes of adult-onset brain disorders in the United States.

**Diagnosis/Cause**	**People Currently Living with Disorder**
Alzheimer's Disease	2,459,000 [2]
Amyotrophic Lateral Sclerosis	36,480 [11,12]
Brain Tumor	401,565 [4]
Epilepsy	2,098,000 [2]
HIV Dementia	328,600 [5,13]
Huntington's Disease	15,611 [7,8,14-17]
Multiple Sclerosis	266,000 [2]
Parkinson's Disease	921,020 [18]
Stroke	5,839,000 [19]
Traumatic Brain Injury	3,170,000 [20]
**Total Estimated Prevalence**	**15,535,276**

By using recent US Census data and current data for prevalence in the United States, an estimate of the number of adults and households affected by brain disorders in the United States was obtained. The results of these estimations are summarized in Table 4.

**Table 4:** Selected population characteristics of the United States.

Total Population	307,066,550 [21]
Total Population 18+	232,403,958 [22]
Total Households	113,568,000 [23]
Total Estimated Adults with Brain Disorders	15,535,276
Percentage of Adult Population Affected by Brain Disorders	6.75%
Percentage of Households Affected by Brain Disorders	13.68%

**Figure 3:**
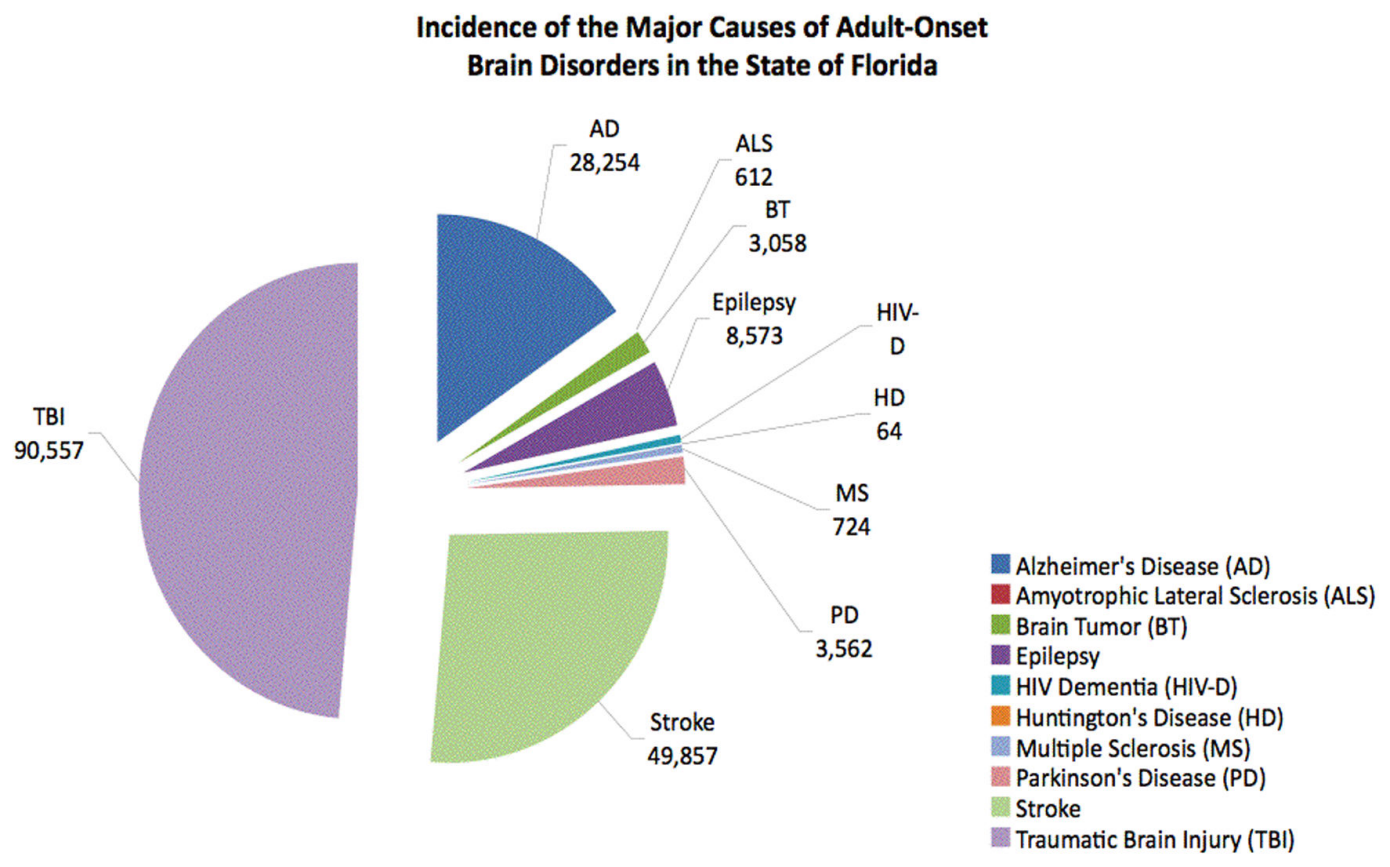
Most current incidence data of adult-onset brain disorders for the state of Florida.

**Figure 4:**
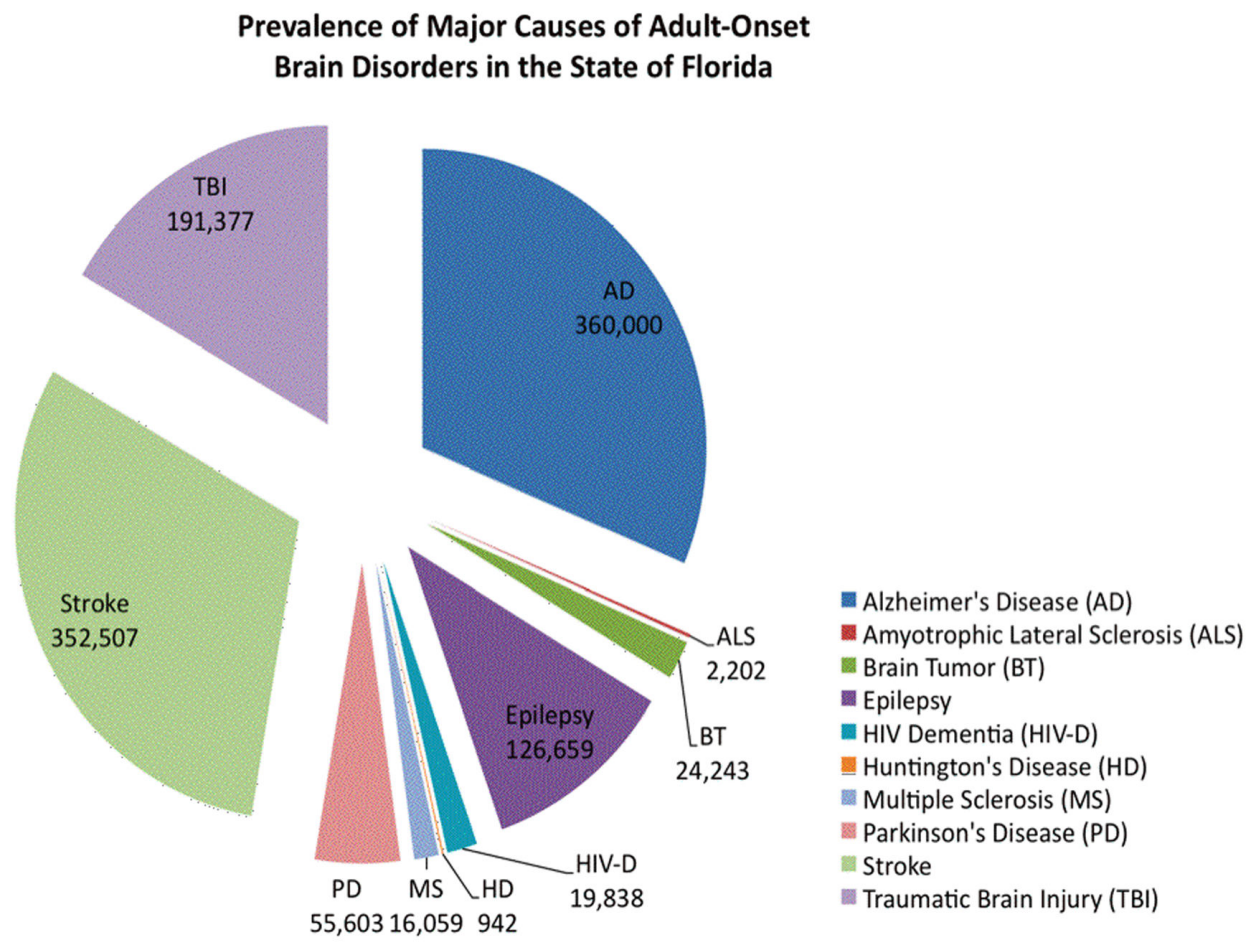
Most current prevalence data of adult-onset brain disorders for the state of Florida.

The incidence and prevalence of brain disorders in the state of Florida were obtained using US Census data for both the nation and the state. The data for incidence and prevalence of the most common adult-onset brain disorders in Florida is summarized in Table 5 and Table 6, respectively. Additionally, incidence data is graphically depicted in Figure 3 and prevalence data is shown in Figure 4. 

**Table 5:** Incidence of the major causes of adult-onset brain disorders in the state of Florida.

**Diagnosis/Cause**	**People Diagnosed Annually**
Alzheimer's Disease	28,254
Amyotrophic Lateral Sclerosis	612
Brain Tumor	3,058
Epilepsy	8,573
HIV Dementia	1,255
Huntington's Disease	64
Multiple Sclerosis	724
Parkinson's Disease	3,562
Stroke	49,857
Traumatic Brain Injury	90,557
**Total Estimated Incidence**	**186,515**

**Table 6:** Prevalence of the major causes of adult-onset brain disorders in the state of Florida.

**Diagnosis/Cause**	**People Diagnosed Annually**
Alzheimer's Disease	360,000 [24]
Amyotrophic Lateral Sclerosis	2,202
Brain Tumor	24,243
Epilepsy	126,659
HIV Dementia	19,838
Huntington's Disease	942
Multiple Sclerosis	16,059
Parkinson's Disease	55,603
Stroke	352,507
Traumatic Brain Injury	191,377
**Total Estimated Prevalence**	**1,149,430**

The number of Floridians and households afflicted with brain disorders was calculated using the most recent Florida population statistics. The percentage of households and adults affected by brain disorders in the state of Florida is described in Table 7.

**Table 7:** Selected population characteristics of the state of Florida.

Total Population	18,537,969 [21]
Total Population 18+	14,324,069 [25]
Total Households	6,337,929 [26]
Total Estimated Adults with Brain Disorders	1,149,430
Percentage of Adult Population Affected by Brain Disorders	8.02%
Percentage of Households Affected by Brain Disorders	18.14%

## Discussion

During the compilation of the prevalence and incidence data for brain disorders in the United States [2-27], it soon became clear that epidemiological data in the United States are extremely scarce for a number of disorders. While Australia and many European countries had current, robust national studies, most of the American data were collected from a small subset of the population. Possible factors contributing to this disparity between the United States and other nations in the literature could involve difficulties in collecting data due to the sheer population of the country and the dynamic nature of the population. Australia and the European nations tend to have national healthcare systems and more rooted populations, which may help explain the discrepancy in the availability of data. Using the adult-onset brain disorder with the highest prevalence, there are 2,459,000 Americans currently living with Alzheimer’s compared with 800,000 people in United Kingdom [28,29], which equate to about 0.8% and 1.3% prevalence rates of this disorder in the two countries, respectively. Additional comparisons of prevalence rates among other brain disorders between the United States and European countries should reveal global disease trends that will require extensive analyses beyond the scope of this study. Clearly future comparative epidemiological studies are warranted. Most often, epidemiological studies in the United States were collected from counties or health maintenance organizations (e.g. Kaiser Permanente) that have access to patient medical records. While the available studies try to reflect the entire United States population, they are incomplete. In order to rectify the lack of epidemiological data, there have been initiatives to create national registries for brain disorders so that more accurate tracking may be possible. Additionally, a move toward national electronic health records may help alleviate the paucity of epidemiological data.

While the lack of data was alarming, the scarcity was not consistent amongst the different brain disorders. Low frequency diseases such as ALS and Huntington’s disease tended to have the weaker data, while high frequency diseases such as stroke, Parkinson’s disease, and Alzheimer’s disease had relatively large data sets. For example, although studies regarding the prevalence of Alzheimer’s disease have been published every five years, it was necessary to go back as far as the 1950s to obtain accurate data for the prevalence of Huntington’s disease. This discrepancy only further emphasizes the need for national disease registries, especially for the rarer conditions.

While the data were limited, enlightening statistics could be deduced regarding adult-onset brain disorders. In a review of historical data, current figures show an increase in brain disorders over the past fifty years, which is entirely expected considering the increasing number of aged individuals in the United States. Furthermore, the data generally agree with many of the most commonly quoted figures of incidence and prevalence. While these quoted statistics are at times completely unsupported by epidemiological studies, it was reassuring to find that the data were not in complete disagreement with the data that are most often divulged to the public. 

Finally, the incidence and prevalence data matched up fairly well. For example, the incidence of Huntington’s disease was found to be 1,053 individuals, while the prevalence was found to be 15,611. The calculated mean duration from these two figures is 14.8 years, which is entirely consistent with the 15.6 average duration of the disease that is mentioned in the literature [2].

The sheer number of Americans with brain disorders was extremely alarming. An estimated 6.75% of the US adult population and 8.02% of the Florida adult population are affected by brain disorders. This discrepancy is not surprising due to the relatively more aged population in the state of Florida. Since nearly 15.5 million individuals are currently affected by brain disorders in the United States, continued research into the causes and treatments of these disorders is of the utmost importance. Furthermore, the fact that nearly 1 out of every 7 households in the United States is affected by brain disorders emphasizes the need for continued education on how to care for those afflicted with brain disorders, especially since these percentages are expected to rise due to the aging baby boomer generation.

Our present estimates of neurological disorders were based on the U.S. Census Bureau. National surveys, such as the National Health Interview Survey (NHIS) also uses the same data collection agency in generating health statistics, such as comparing male Veterans versus nonVeterans. Moreover, when NHIS addresses the issue of multiple chronic conditions, the focus is on hypertension, diabetes, heart disease, and cancer with neurological disorders largely relegated to stroke. Another survey, called the National Health and Nutrition Examination Survey (NHANES), focuses on assessment of US health and nutritional status. These NHANES statistics generally summarize caloric intake from food and diet in adult and young Americans, and when diseases are examined, the focus is on obesity and hypertension, which obviously are of important health- and nutrition-related diseases. Finally, national insurance databases are also a good source for general health statistics, but found that many of these health insurance coverage surveys utilize the NHIS data, which as we noted above were based on U.S. Census Bureau data that we used in the present survey. Unfortunately, just like the NHIS and the NHANES, these insurance databases (e.g., Medicaid and Medicare) do not report prevalence of neurological disorder estimates. Accordingly, our present survey reports timely and accurate prevalence rates of neurological disorders, which are not covered by NHIS, NHANES or national insurance statistic surveys. 

We acknowledge the following limitations of this study. The current formula used for calculating the percentage of US households affected by brain disorders did not take into account the numbers of households with more than one case of brain disorder, which will require modifications (e.g., 100*[1-(1-[% of adults with brain disorders])^[average size of household])]). We also caution that our focus is adult neurological disorders, requiring new calculations for children neurological disorders. Moreover, the actual prevalence of certain brain disorders, like Huntington’s disease, may be higher than that reported in the literature. This is the overarching objective of our study, in that there seems to be a disconnect between the reported literature and the actual prevalence numbers. Case in point is that many of the numbers reported are based on literature published at least one year earlier and in most cases many years earlier therefore not capturing the current numbers. Our proposed formula employs the prevailing household census thus providing more updated numbers approximating the current national health system numbers. Finally, our epidemiological study did not examine mental disorders, which will be subsequently analyzed.

Because government funding and other national research foundations, in part, based their research grant funding appropriations on the prevalence of specific brain disorders, the reporting of actual prevalence numbers is pivotal to guiding the future research direction that will eventually lead to clinical applications, thereby directly affecting the health care status. The availability of recently updated and accurate epidemiological data is likely to serve as key guidance figures for basic science and clinical research, as well as health care. In this regard, at least in the US, stroke, epilepsy, Alzheimer’s disease, and a surging increase in the number of cases of traumatic brain injury represent disease indications that would urgently merit from reporting of precise epidemiological data. The successful solicitation of public awareness and governmental support will benefit from a collective discussion of brain disorders supported by systematic and objective numbers of prevalence as being reported here. The precedent success garnered by the cancer field via a unified approach in getting the attention of both public and government sectors should be considered.

## Conclusions

The aggregation of the most current and well-supported data regarding brain disorders will be a powerful tool for both clinicians and researchers. As presented, this study allows for a convenient comparison of the most common adult-onset brain disorders. This study fills a glaring void in the available literature, and this clear, logical format will only become more useful as the epidemiological data improve with the concurrent aging of the American population.

## Supporting Information

Checklist S1 **MOOSE Checklist.**
(DOCX)
