# Supplementary material for: Epidemiological Survey-Based Formulae to Approximate Incidence and Prevalence of Neurological Disorders in the United States: a Meta-Analysis
Source: PLoS One. 2013 Oct 24;8(10):e78490. doi: 10.1371/journal.pone.0078490 (PMC3812041; doi:10.1371/journal.pone.0078490)
Supplement: Checklist S1 — MOOSE Checklist. (DOCX) [file pone.0078490.s001.docx]

Supporting Information S1: **MOOSE Checklist for Epidemiological Survey-Based Formulae to Approximate Incidence and Prevalence of Neurological Disorders in the United States: a Meta-analysis**

Cesar V. Borlongan, Ph.D.

Department of Neurosurgery and Brain Repair, University of South Florida

Jack Burns, M.S.

Department of Neurosurgery and Brain Repair, University of South Florida

Naoki Tajiri, Ph.D.

Department of Neurosurgery and Brain Repair, University of South Florida

Christine E. Stahl, M.D.

Department of Neurosurgery and Brain Repair, University of South Florida

Nathan L. Weinbren, H.S.

Department of Neurosurgery and Brain Repair, University of South Florida

Hideki Shojo, Ph.D.

Department of Neurosurgery and Brain Repair, University of South Florida

Paul R. Sanberg, Ph.D.

Department of Neurosurgery and Brain Repair, University of South Florida

Dwaine F. Emerich, Ph.D.

Department of Neurosurgery and Brain Repair, University of South Florida

Yuji Kaneko, Ph.D.

Department of Neurosurgery and Brain Repair, University of South Florida

Harry R. van Loveren, M.D.

Department of Neurosurgery and Brain Repair, University of South Florida

Corresponding Author :

Cesar V. Borlongan

Address: 12901 Bruce B. Downs Blvd. Department of Neurosurgery and Brain Repair, University of South Florida, Tampa, FL 33612

E-mail: cborlong@health.usf.edu

Phone: 813-974-3154

Fax: 813-974-3078

| **Criteria** | | **Brief description of how the criteria were handled in the meta-analysis** |
| --- | --- | --- |
| **Reporting of background should include** | |  |
| √ | Problem definition | A brain disorder is an ailment that impairs cognitive and motor functions. These disorders include, but are not limited to, amyotrophic lateral sclerosis (ALS), Alzheimer’s disease, brain tumor, epilepsy, HIV dementia, Huntington’s disease, multiple sclerosis, Parkinson’s disease, stroke, and traumatic brain injury. Age-related brain disorders are predicted to increase, yet there are no collected incidence and prevalence data. |
| √ | Hypothesis statement | Census data will approximate the incidence of neurological disorders. |
| √ | Description of study outcomes | Census data closely approximated incidence. |
| √ | Type of exposure or intervention used | No exposure or intervention used |
| √ | Type of study designs used | We included published census data. |
| √ | Study population | No restrictions |
| **Reporting of search strategy should include** | |  |
| √ | Qualifications of searchers | The credentials of the authors are listed above. All searchers were trained by seasoned investigators with an M.D. or a Ph.D. |
| √ | Search strategy, including time period included in the synthesis and keywords | Using PubMed and Google, keywords used were census, incidence, prevalence, neurological disorders, brain, and United States. Keywords were searched as stand-alone words or in combinations. |
| √ | Databases and registries searched | PubMed and Google |
| √ | Search software used, name and version, including special features | Windows Internet Explorer 9 |
| √ | Use of hand searching | No hand searches |
| √ | List of citations located and those excluded, including justifications | When searching with the initial keywords listed above, 673 articles were found on PubMed. When specifications were made from brain-related neurological disorders, 81 articles were found, indicating an exclusion of 592 articles. When United States was added to the search queue, 51 more articles were excluded. When specification was made for age-related brain disorders, the final count of studies was 19. Studies revealing only numerical data were used.  When the same process was performed in Google, and the search was limited to government-affiliated websites, only a handful of articles were generated and were subsequently used in our study. |
| √ | Method of addressing articles published in languages other than English | Though there was no language restriction placed, only English articles were found. |
| √ | Method of handling abstracts and unpublished studies | No abstracts and unpublished studies were used. |
| √ | Description of any contact with authors | No contact with the authors was made. |
| **Reporting of methods should include** | |  |
| √ | Description of relevance or appropriateness of studies assembled for assessing the hypothesis to be tested | Table 1 |
| √ | Rationale for the selection and coding of data | All Data involving neurological disorders were used to find the incidence and prevalence. All census data were taken for a control for the population. |
| √ | Assessment of confounding | None |
| √ | Assessment of study quality, including blinding of quality assessors; stratification or regression on possible predictors of study results | Study quality was not assessed since our study was limited to census numbers released by government-affiliated websites. |
| √ | Assessment of heterogeneity | Heterogeneity was not assessed since our study was limited to census numbers released by government-affiliated websites. |
| √ | Description of statistical methods in sufficient detail to be replicated | Statistical methods, with detailed formulae used to calculate incidence and prevalence of neurological disorders, were provided in in our study. |
| √ | Provision of appropriate tables and graphics | See Figures 1-4 and Tables 2-7 |
| **Reporting of results should include** | |  |
| √ | Graph summarizing individual study estimates and overall estimate | Figure 1-4 |
| √ | Table giving descriptive information for each study included | Table 1 |
| √ | Results of sensitivity testing | Sensitivity of the data is limited to the available census data. |
| √ | Indication of statistical uncertainty of findings | Statistical uncertainty is also limited to the available census data. |
| **Reporting of discussion should include** | |  |
| √ | Quantitative assessment of bias |  |
| √ | Justification for exclusion | Data were excluded if they were not brain-related neurological disorders, from or about the United States, or did not contain numerical statistics. |
| √ | Assessment of quality of included studies | Study quality was not assessed since our study was limited to census numbers released by government-affiliated websites. |
| **Reporting of conclusions should include** | |  |
| √ | Consideration of alternative explanations for observed results | The last United States census was in 2009. Since then, there may be slight fluctuations in statistics on population sizes. However, these are miniscule at best and are easily corrected with population growth analysis. |
| √ | Generalization of the conclusions | Formulae are able to evaluate census data |
| √ | Guidelines for future research | Use of these formulae to find the epidemiology of other diseases. |
| √ | Disclosure of funding source | From page 1:  **Source of support:** Financial support for this study was through the University of South Florida Department of Neurosurgery and Brain Repair funds. CVB is funded by the James and Esther King Biomedical Research Foundation 1KG01-33966, NIH [5U01NS055914-04](https://commons.era.nih.gov/commons/genericStatus.do?actionRole=nonPI&applID=7940916&uhf-token=tEjSzyKo0GhJi%2BTX76zgTe6lyEY%3D) and NIH [1R01NS071956-01A1](https://commons.era.nih.gov/commons/genericStatus.do?actionRole=nonPI&applID=8228397&uhf-token=npeOtwcorZY6yDpWSKoi8IF%2BrDs%3D). |
